# Wine glasses and hourglasses: Non-adaptive complexity of vesicle traffic in microbial eukaryotes

**DOI:** 10.1016/j.molbiopara.2016.03.006

**Published:** 2016

**Authors:** Somya Mani, Mukund Thattai

**Affiliations:** Simons Centre for the Study of Living Machines, National Centre for Biological Sciences, Tata Institute of Fundamental Research, UAS-GKVK Campus, Bellary Road, Bangalore 560065, India

**Keywords:** Eukaryote evolution, Organelles, Compartments, Vesicle traffic, Boolean models

## Abstract

Microbial eukaryotes present a stunning diversity of endomembrane organization. From specialized secretory organelles such as the rhoptries and micronemes of apicomplexans, to peroxisome-derived metabolic compartments such as the glycosomes of kinetoplastids, different microbial taxa have explored different solutions to the compartmentalization and processing of cargo. The basic secretory and endocytic system, comprising the ER, Golgi, endosomes, and plasma membrane, as well as diverse taxon-specific specialized endomembrane organelles, are coupled by a complex network of cargo transport via vesicle traffic. It is tempting to connect form to function, ascribing biochemical roles to each compartment and vesicle of such a system. Here we argue that traffic systems of high complexity could arise through non-adaptive mechanisms via purely physical constraints, and subsequently be exapted for various taxon-specific functions. Our argument is based on a Boolean mathematical model of vesicle traffic: we specify rules of how compartments exchange vesicles; these rules then generate hypothetical cells with different types of endomembrane organization. Though one could imagine a large number of hypothetical vesicle traffic systems, very few of these are consistent with molecular interactions. Such molecular constraints are the bottleneck of a metaphorical hourglass, and the rules that make it through the bottleneck are expected to generate cells with many special properties. Sampling at random from among such rules represents an evolutionary null hypothesis: any properties of the resulting cells must be non-adaptive. We show by example that vesicle traffic systems generated in this random manner are reminiscent of the complex trafficking apparatus of real cells.

## Highlights

• We are motivated by the diversity of vesicle traffic systems in microbial parasites.
• We present a mathematical model of vesicle traffic in a manner accessible to a broad audience.
• We show that many complex features of vesicle traffic systems arise spontaneously due to molecular interactions.
• Traffic features such as compartmental maturation might arise non-adaptively and later be selected for function.

## 1 Introduction

The intracellular organization of all eukaryotic cells are variations on a basic theme of membrane compartmentalization. Within this theme there is remarkable diversity across the eukaryotic tree of life [1], [2]. Many microbial eukaryotes, especially parasites with extreme lifestyle adaptations, have particularly exotic endomembrane systems. Some of this diversity is contributed by taxon-specific modifications of endosymbionts including plastids and mitochondria [3], [4], but most organelles have a non-endosymbiotic origin, and are part of a dynamic endomembrane system. Peroxisomes play varied metabolic roles across eukaryotes, and have evolved to contain distinct types of metabolic machinery in different taxa [5]. Particularly divergent examples of peroxisomes are the glycosomes of kinetoplastids such as Trypanosoma and Leishmania, which contain an extended glycolytic pathway [6], [7]. The post-Golgi pathway is extensively modified in many organisms, especially parasites, adapted for secretion. For example, the rhoptries, micronemes and dense granules of the apicomplexans Toxoplasma and Plasmodium [8], [9], [10], [11], [12] are specialized secretory organelles essential for host cell invasion. Apart from the core secretory and endocytic pathways, these embellishments do not fit any simple formula: each new species presents new surprises. Parasites provide particularly well-studied instances of complex traffic systems, but this pattern repeats across the eukaryotes. This complexity is the starting point for our discussion. Should we think of it as arising from niche-specific selective pressures? Must traffic systems be wired up in intricate ways in order to perform particular biochemical functions?

To understand the mechanistic and evolutionary origins of these diverse systems, it is important to study not just the endomembrane organelles, but also the processes by which proteins and other cargo are sorted and transported between them. The endomembrane system is dynamic, and compartment compositions are determined as the outcome of the exchange of material in vesicles. [13]. Vesicle traffic is driven by a common molecular repertoire across eukaryotes [14]. At its center are the twin processes of vesicle budding and fusion. Coatomers and adaptors [15], [16] load specific subsets of cargo from the source compartment [17] onto budding vesicles. By cargo sorting, multiple vesicles of distinct compositions can be generated from the same source compartment [18], [19]. Tethers and SNARE proteins drive the fusion of these vesicles into specific target compartments [20], [21], [22]. ARF and Rab GTPases [23], [24] regulate multiple steps of vesicle budding and fusion, ensuring the correct specificity and timing of events.

Compartment and vesicle behavior are governed by transmembrane molecules (such as receptors, SNAREs and lipid varieties) in conjunction with cytoplasmic molecules (such as ARFs, Rabs, coatomers and tethers). The very process of budding or fusing a vesicle requires the vesicle to carry certain transmembrane molecules, and necessarily causes changes to the compositions of source and target compartments. Therefore, it is impossible to study any one pathway of a vesicle traffic system without considering the flow of material across the entire system. Perturbations at any point in such an interconnected network can have cascading effects throughout the cell. If the state of a cell appears invariant over time, this is only because a large number of budding and fusion events balance one another out.

In this context, mathematical modeling has proved useful in connecting basic molecular events, such as budding and fusion, to dynamic processes, such as cargo transport, and finally to the compartmental compositions of the endomembrane system [25], [26], [27], [28], [29], [30]. These models require the specification of a large number of parameters. Typically, many of these parameter values are estimated indirectly, and very few are obtained from prior experimental measurements. Such analyses can be applied to study a specific system of interest, but do not provide a natural framework to study diversity.

It would be useful to be able to “try out” the behavior of millions of hypothetical cells over millions of possible parameter combinations, and see the spectrum of resulting cellular properties. For this to be possible the model itself has to be made very simple. This is precisely the goal of so-called Boolean models, where the state of the system is described by a series of 1s and 0s representing the presence and absence of molecular components. Boolean models are efficient to simulate on a computer, and therefore allow the rapid exploration of a large parameter space. Such approaches have been extremely useful in revealing the inner workings of gene regulatory networks and signaling networks [31], [32], [33]. Here we present the outlines of such a Boolean model when applied to vesicle traffic. Our discussion is pedagogical: we consider specific examples in detail, and avoid focusing on mathematical derivations. Our goal is to introduce the reader to a new way of thinking about what models are and what they can do. We propose that Boolean models can be used to understand the vesicle traffic system and its diversity across eukaryotes.

## 2 Methods

### 2.1 Models of vesicle traffic: complex to simple

We want to model a cell containing multiple distinct compartments exchanging molecules via vesicles. The basic question is: given some underlying model and its parameters, what are the different compartments that arise and how are they connected? The most general traffic model would include the spatial location, shape, size and molecular compositions of these compartments, and would account for stochasticity in vesicle budding and fusion events. As a reasonable simplification we could ignore the spatial location and shape of compartments, and focus instead on their size, composition, and connectivity alone. Models that focus on size distributions allow for a continuum of structures from transport vesicles to large compartments, and account for fission and fusion of all such structures [26]. Many models focus on compartments and the complex rules of molecular specificity, but do not treat transport vesicles explicitly and do not account for inter-vesicle fusion [25], [28], [29]. In such models, compartment compositions are specified by the concentrations of various molecules, and vesicle traffic is represented by a series of ordinary differential equations. This approach has been applied to consider the detailed case of vesicle exchange between two compartments [25], [28] but can be extended to more complex many-compartment traffic systems [29]. Adding stochastic processes to these formulations puts further constraints on the conditions under which compositionally distinct compartments can arise [28], [30].

In general, detailed models are useful for comparisons with cell biological data in specific cases. However, they require the specification of a large number of parameters and therefore can only be studied under limited parameter combinations. Here we use a Boolean framework, which ignores size, shape, and location, and focuses on composition alone. We assume that the behavior of a compartment depends on whether it contains various molecular components in sufficient amounts. By simplifying the model itself, we gain the ability to sample over a large space of possible rules. In Section 2.2 we describe how to set up and run a Boolean vesicle traffic model. In Section 3.1 we show the results of running such a model for many randomly chosen rules.

### 2.2 Ingredients of a Boolean model of vesicle traffic

**Fig. 1:**
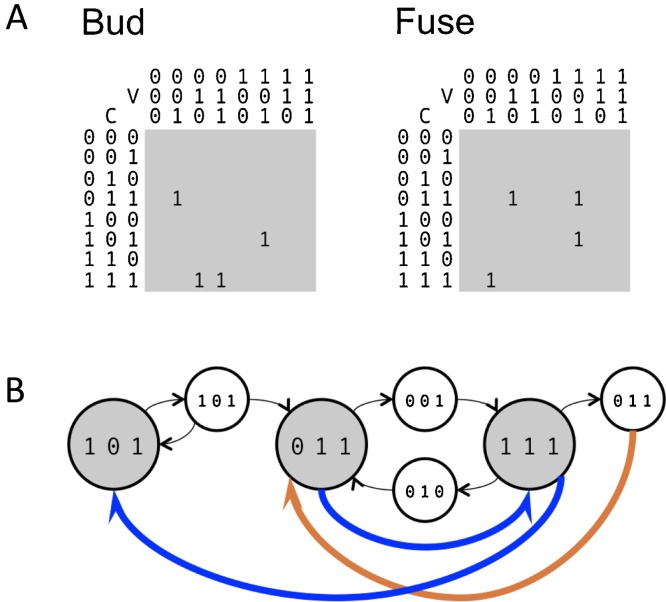
The Boolean vesicle traffic model. (A) For *N* = 3 active molecular labels, there are $2^{N}=8$ possible binary compartment and vesicle types. The 8×8 budding Table shows which compartments (rows) can generate which vesicles (columns), and similarly the 8×8 fusion Table shows which vesicles can fuse to which compartments. (B) The steady state of a system defined by the budding and fusion tables from A, involving three compartments and four vesicle types. Compartments can undergo compositional maturation (bold blue arrow from compartment to compartment); vesicles can undergo homotypic fusion to generate a new compartment (bold brown arrow from vesicle to compartment). (For interpretation of the references to colour in this figure legend, the reader is referred to the web version of this article.)

To begin this process, we must decide which set of molecular types will be relevant to include in the model. We could list out all the distinct homologs of SNARE subunits, all signaling lipids, transmembrane receptors, and so on. Suppose at the end of this process we are left with *N* types of molecules which might influence vesicle budding and fusion. We will refer to these molecules as active labels, to distinguish them from the passive cargo contained within vesicles. We then summarize the composition of a compartment or a vesicle with a list of 1s and 0s of length *N*. Every 1 such a list means that the corresponding compartment contains a high concentration of the corresponding molecular label. Let us consider a very simple case in which three different types of molecules are spread across a vesicle traffic system. Therefore *N* = 3, and each compartment is described by a binary list (Fig. 1A) such as [101] (the first and third molecular types are present in high amounts, the second one is absent) or [111] (all molecular types are present in high amounts). There could be many compartments with the same binary composition, just as there could be many endosomes or many *cis*-Golgi cisternae. We imagine that the entire set of such compartments comprise an organelle. The “state” of the cell as a whole is described by a list of lists, namely by listing the compositions of all the compartments that happen to be present. For example, the cell in Fig. 1B has three distinct compartments, and is described by the list of lists [101], [011], [111]. Any system will start off in some state known as the “initial condition,” which specifies how the molecules are distributed among the available compartments at the initial timepoint. As time proceeds, the compositions of the compartments will change due to exchange via vesicles.

We next describe the rules of vesicle budding and fusion that cause these compositional changes. Rather than presenting an abstract formulation, our discussion is based on the example of Fig. 1. Assuming the “rules” are those shown in Fig. 1A, and the “initial condition” is that shown in Fig. 1B, we describe step-by-step the resulting events and the specific changes they cause. Transport vesicles are much smaller than organellar compartments, but as long as we keep track of whether we are discussing compartments or vesicles, we can use the same type of binary labels for both. Thus the compartment of composition [011] buds out a vesicle of composition [001], through some process of coatomer recruitment and subsequent interaction of molecular labels and adaptors. This vesicle fuses into a target compartment of composition [111]; it does not fuse into any of the other compartments that happen to be present, due to some underlying molecular specificity provided by Rabs, tethers and SNAREs. Extending this idea, we must list the “rules” implied by the underlying molecular interactions: all the possibilities by which vesicles are allowed to bud out of source compartments or fuse into target compartments. This is shown in Fig. 1A as a budding table and a fusion table. The specific locations of the 1s in these tables are determined by specific knowledge of how ARFs, coatomers and adaptors (for budding) and Rabs, tethers and SNAREs (for fusion) interact. For example, the number of different adaptor/coatomer complexes in a cell limits the maximum number of distinct vesicle types that a single source compartment can generate. If this information is not known or only partially known, we can still place 1s and 0s at random and sample over a large number of possible budding and fusion rules.

Having described the specification of the “state” in terms of compartment and vesicle compositions, and “rules” in terms of the budding and fusion tables, we must consider the “dynamics”: how the constant budding out and fusing in of vesicles changes compartment compositions. Consider the compartment [101] which is simultaneously budding out and fusing with vesicles of the same composition (vesicle [101]). Since loss and gain are balanced, the composition of this compartment does not change. The same argument must be applied to each individual label type in turn. Compartment [111] is neither gaining nor losing the first label type, and it is both gaining (via vesicle [001]) and losing (via vesicle [011]) the third label type. However, it is losing the second label type (via vesicles [011] and [010]) and this loss is not balanced by any gain. Therefore, as time proceeds and a steady stream of budding and fusion events occur, the first and third label types remain in balance, but the second label type is lost. Compartment [111] therefore “becomes” compartment [101], as represented by the bold blue arrow. By the same logic, compartment [011] “becomes” compartment [111]. This is the equivalent to the phenomenon of “compartmental maturation” seen in the progression of Golgi cisternae compositions [34], [35], [36], [37], the early-to-late endosome transition [38], and the maturation of precursor compartments to rhoptries in apicomplexans [8], [9], [10], [11], [12]. During maturation the compartment remains as an identifiable membrane-enclosed entity, but its composition changes over time.

A vesicle type that has nowhere to fuse, as with vesicle [011] budding out of compartment [111], will accumulate in the cytoplasm. In our model, we imagine that once sufficient time has passed, such vesicles undergo homotypic fusion to seed a new compartment, as is speculated to occur in the ER-Golgi intermediate compartment (ERGIC) [37]. This process is essential to compensate for the constant maturation of compartment compositions. In this way, the pool of vesicles of composition [011] undergoes homotypic fusion to generate a new compartment of composition [011], as represented by a bold brown arrow. This balances out the loss of the existing compartment [011] due to maturation. Importantly, for the cell shown in Fig. 1B the overall state of the cell does not change even though the system is extremely dynamic. Either molecular gain and loss are balanced, or the maturation of a compartment is compensated for by the creation of a new compartment. This is known as a “steady state.”

Now that we’ve described how the model actually works for a specific example, we are ready to consider the general case. Suppose we are provided with some budding and fusion tables. Keeping these rules fixed, we can start a cell off in many different initial conditions. A random initial condition is unlikely to itself be a steady state, we must run the system for a large number of timesteps and see which steady state it converges to. We can repeat this procedure for different budding and fusion tables. In this way, we can figure out which types of rules give which types of steady states. The key question is: how do we fill in the budding and fusion tables in the first place? Given all the details of molecular interactions – which combinations of molecules recruit which coatomers, how molecules interact with adaptors for vesicle loading, and which combinations of molecules activate or inhibit fusion – we could fill in the 1s and 0s of the budding and fusion tables directly. In practice such information is never comprehensive. Alternatively, we could fill in 1s and 0s at random and simulate the resulting model, and then repeat this for millions of random tables. This is the approach we use here. Any properties that occur with high frequency in the resulting cellular steady states are essentially independent of the specific location of 1s and 0s in the random tables.

## 3 Results and discussion

### 3.1 Special properties of randomly generated cells

**Fig. 2:**
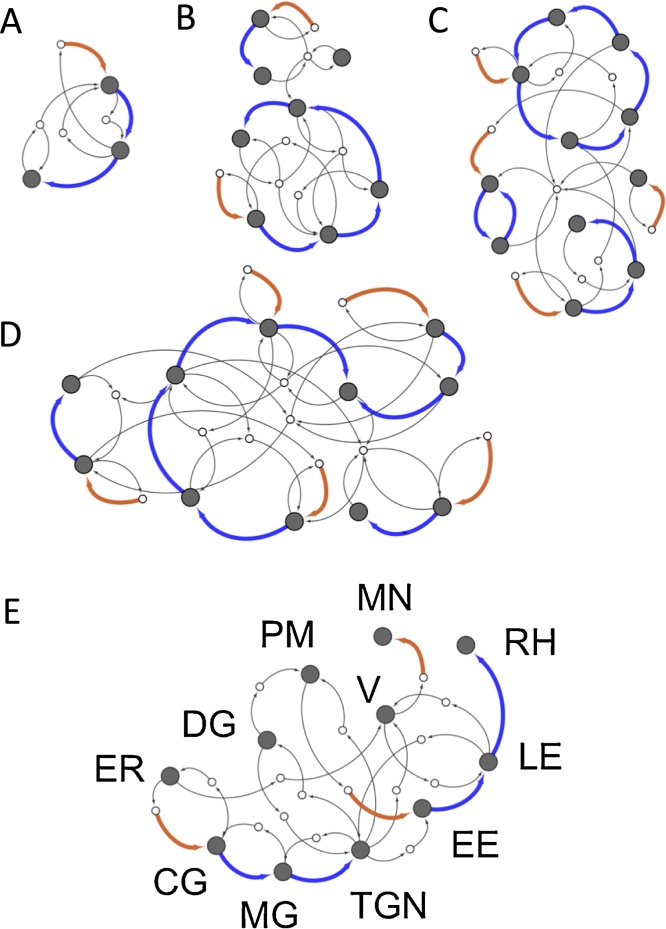
Steady states of random budding and fusion rules. Compartments are gray circles, vesicles are white circles. Light arrows show vesicle budding and fusion. Bold brown arrows show the creation of a compartment by homotypic fusion of a vesicle pool. Bold blue arrows show compartmental maturation. (A) The steady state for the *N* = 3 label system shown in Fig. 1B. (B–D) Steady states for randomly generated budding and fusion rules with *N* = 7 label types. (E) Schematic vesicle traffic system of Toxoplasma gondii. The secretory system is adapted from Ref. [10], the endocytic system is adapted from Ref. [8]. The precise nature of endocytosis and its connection with the TGN and early endosomes is not known. This figure is meant to be representative of the complexity of the system, but not authoratative regarding particular connections. More details about can be found in Ref. [12]. ER: endoplasmic reticulum. CG: *cis*-Golgi. MG: medial-Golgi. TGN: *trans*-Golgi network. PM: plasma membrane. EE: early endosome. LE: late endosome. V: vacuole. DG: dense granule. RH: rhoptry. MN: microneme. (For interpretation of the references to colour in this figure legend, the reader is referred to the web version of this article.)

Molecular interactions determine the rules. These rules, *in turn*, determine the steady states and their properties. In the absence of detailed molecular information, we sample over a large number of rules. Here we present a few concrete examples of this procedure. In Fig. 2 we show the steady state cells which result from a series of random budding and fusion rules, for the case of *N* = 7 label types. For comparison (Fig. 2E), we have shown the schematic traffic system of Toxoplasma gondii [8], [9], [10], [11], [12]. What immediately jumps out from these figures is the high degree of structure and visual complexity of the random cells, similar to that of a real vesicle traffic system. We see chains and cycles of compartmental maturation (blue arrows), seeded by homotypic vesicle fusion (brown arrows). We see clusters of vesicle exchange between centralized compartments of high connectivity and peripheral compartments of low connectivity. There are many other special properties one could imagine these traffic networks to have, and it is possible to test for them statistically [39]. A full analysis of this type will be published separately as a follow-on paper, providing a more mathematical treatment. In the following section we provide a heuristic explanation for why such complex properties might be expected to arise spontaneously for a vesicle traffic system.

### 3.2 Enumerating states and rules

Our argument is based on counting how many possible states a cell can be in, and how many possible rules could determine its dynamics. We repeatedly apply a simple mathematical formula: if we want to assemble a collection of *N* distinct entities, each of which could be present or absent, there are $2^{N}$ ways to do this. For example, for *N* = 3 label types, there are $2^{N}=8$ possible compartment or vesicle types, and the budding and fusion tables are both 8×8 tables with 128 elements in total. If each of these elements can be independently specified, there are $2^{128}=2^{2^{2N+1}}$ possible vesicle traffic rules (repeated exponentials should be evaluated from right to left).

**Fig. 3:**
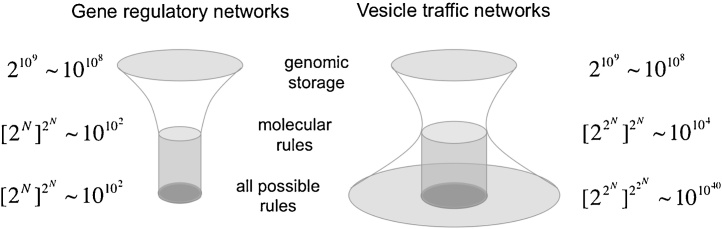
Wine glasses and hourglasses: the space of rules of gene regulatory networks and vesicle traffic networks. The top layer represents the available genomic storage space, for a billion-base genome. The middle layer represents the set of physically realizable rules through molecular interactions. The bottom layer represents the set of all possible dynamical rules. The powers of two indicate the calculated number of possibilites at each layer (Section 3.2). The powers of ten indicate approximate values for a system containing *N* = 7 genes or active label types. Exponentials are evaluated from the right, working leftwards, so things get very big very quickly. For example, $10^{10^{2}}$ is the same as $10^{100}$ which is a 1 followed by 100 zeros. These numbers are only presented to show the awesome scale of available possibilities.

For the purposes of discussion, it is useful to compare vesicle traffic networks (VTNs) with gene regulatory networks (GRNs) [31], each having *N* label types or regulated genes (Fig. 3). We indicate the number of possible states and rules by *S* and *R*, respectively. The state of a GRN is specified by listing the set of genes that are transcriptionally active at any timepoint. Since there are *N* genes, each of which could be on or off, the whole system is specified by a binary list of length *N*, and there are $S_{GRN}=2^{N}$ possible transcriptional states. The state of a VTN is specified by listing the set of distinct compartments present in a cell at any timepoint. Since there are $2^{N}$ possible compartment types, each of which could be present or absent in the cell, there are $S_{VTN}=2^{2^{N}}$ cell types.

Once a GRN or VTN is placed in some initial condition, the dynamical rules determine how the state of the system changes over time. If we consider a deterministic system which updates in discrete timesteps, then the dynamics can be written as a simple table: the first column lists every possible state at time *t*, ordered from 1 to *S*; the second column lists the updated value of these states at time t+1. Each of the *S* entries of the second column can be independently specified from among all *S* possibilities. There are therefore nearly $S^{S}$ possible ways to describe the dynamics of a system. (The “nearly” arises because some of these rules might be the same as others, only that molecular identities are switched.)

In general, not all possible dynamical rules can be realized through physical interactions of molecules. For a GRN, suppose every gene encodes a transcription factor that could bind to the upstream regulatory region of any other gene, including itself. We know there are $S_{GRN}=2^{N}$ ways in which transcription factors may be present or absent at any timepoint. For each of these options, we must specify whether some gene of interest is on or off at the next time point due to transcriptional regulation. There are $2^{S_{GRN}}=2^{2^{N}}$ ways to do this for any given gene, and therefore ${\left[2^{2^{N}}\right]}^{N}={\left[2^{N}\right]}^{2^{N}}$ possible ways to do this over all *N* genes. The size of the rule space is therefore $R_{GRN}={\left[S_{GRN}\right]}^{S_{GRN}}$. All possible dynamical rules can be realized, assuming each upstream regulatory region is sufficiently complex. For a VTN, the molecular rules are specified by filling in 1’s and 0’s into the $2^{N}\times 2^{N}$ budding and fusion tables. There are about $2^{2N}$ elements to independently specify, which can be done in $2^{2^{2N}}$ possible ways. Therefore $R_{VTN}∼{\left[S_{VTN}\right]}^{\log_{2}S_{VTN}}$ which is much less than ${\left[S_{VTN}\right]}^{S_{VTN}}$.

There are three takeaway messages from this analysis (Fig. 3). First, the set of states of a VTN is enormously larger than that of a GRN with the same number of molecular components. This is precisely because, for a VTN, those components could be spread in many ways across many compartments. Second, all possible types of rules of a GRN can in principle be achieved using sufficiently complex molecules. The behavior of a GRN is therefore not constrained. Third, the set of rules of a VTN that can be achieved through molecular interactions is a vanishingly small fraction of the set of all possible rules we might expect for a system with so many states. Molecular rules of a VTN therefore represent a very special subset of all possible rules. This last point is expanded in the following section.

### 3.3 Wine glasses and hourglasses

It is useful to think of a vesicle traffic network in three layers (Fig. 3). The top layer is the genomic storage space which actually encodes all the relevant proteins and regulatory elements. The middle layer represents the space of physically realizable rules, which emerge through the interactions of these molecules. The bottom layer represents the abstract space of all possible dynamical rules. Gene regulatory networks form a wine glass: a large genomic storage space collapses onto a small set of molecular rules, but these fully cover all possible dynamical rules. In contrast, vesicle traffic networks form an hourglass: the set of molecular rules is the bottleneck, smaller than the storage space and much smaller than the set of possible dynamical rules. There are many hypothetical vesicle traffic rules we could imagine, and there is sufficient space to encode such rules in the genome, but most of them will not be consistent with any system that can arise through molecular interactions.

The evolution of intracellular traffic does not proceed through the direct addition of new vesicles and compartments to existing systems: evolution operates in the top layer, as random mutations sample the space of possible genomes. Most such genomes are not viable, but some result in functional traffic rules through molecular interactions. These *rules* then generate the steady-state traffic system, with some set of compartments interconnected by vesicles. We cannot fully know the complexities of mutation, selection and drift, nor the sequence-function relationship of proteins. Nevertheless, we can directly analyze the middle layer of rules. By sampling molecular rules at random, we are testing an evolutionary null hypothesis. Due to the hourglass nature of vesicle traffic, rule-generated traffic systems have many properties which would be unexpected had we sampled the *bottom* layer at random. The requirement that molecules must be transported in vesicles means that traffic systems have a large number of constraints, and consequently a large number of special properties not expected under random compartmental dynamics.

## 4 Conclusion

### 4.1 The ubiquity of compartmental maturation

Compartmental maturation has emerged as a recurring theme in cell biology. In some eukaryotic species including metazoans, the cisternae of the Golgi apparatus undergo compositional maturation [34], [35], [36], [37]. This allows the processing of large cargo which cannot fit in small transport vesicles [36], [37]. The early endosomal compartments of many eukaryotes undergo a series of compositional changes before finally fusing with the lysosome [38]. From these diverse examples it might appear that maturation is a specifically selected process driven by specialized molecular machinery. We would argue the opposite: chains of compartmental maturation arise in randomly generated vesicle traffic systems through pure mass balance constraints, in the absence of selective pressure (Fig. 2). Indeed, it would be surprising if real cells did *not* show evidence of maturation. Of course, once mutation has provided an embellished traffic system, mistargeting of enzymes and cargo into new pathways could provide a substrate for selection [40]. Thus, a complex structure that initially arose non-adaptively could be exapted for new function.

For example: more so than free-living organisms, parasites are engaged in a constant arms race with their hosts. For Toxoplasma, rhoptry-secreted factors are a key determinant of virulence and thus under constant selective pressure [41]. It is striking that the secretory system of Toxoplasma appears to have “repurposed” the endosomal maturation system for the production of rhoptries [11]. Compartmental maturation is an evolutionarily available source of organellar variation, providing a rich substrate upon which selection can act to modify parasitic secretory pathways.

### 4.2 Models in cell biology

We hope this discussion serves to highlight the utility of simple models, which can help to identify general properties of biological systems even when they do not contain enough detail to be predictive in specific cases. For most biological systems, even under the most optimistic scenarios, there will never be enough information to constrain a complex model, and most of its parameters will be mere guesses. In this situation of incomplete information, the modeler must take a systematic approach.

First: decide the level of detail at which we would like to describe some phenomenon of interest. In this case, we are interested in the inter-organelle connectivity of a vesicle traffic network, not in features such as spatial location. One reason this is a good choice is that information about connectivity can be obtained from genetic and cell-biological measurements. Second: determine the least number of assumptions necessary to reproduce known features of the system at the chosen level of detail. If the model fails at this stage, change the assumptions. For example, the movement of single SNARE proteins is beyond the purview of the model; but compartmental maturation is fair game. If we had found that our vesicle traffic model could not account for maturation, a common feature of parasite traffic, it would have signified a serious error and we would have had to return to the drawing board. Third: once the model passes the basic check of reproducing a known set of features, find out what else it predicts. This is more an art than a science.

Let us subject our model to the test. What has it taught us? One important contribution is to rule out previously proposed vesicle traffic mechanisms whose only justification was to explain compartmental maturation. For example, biophysical models have invoked a protein gradient caused by rapid SNARE decay as a mechanism to set up Golgi maturation [42]. We have shown that no assumptions other than the basic mechanisms of vesicle budding and fusion are needed for maturation to arise. Unless some independent evidence exists for rapid SNARE decay, the observation of maturation on its own is not enough to justify such a hypothesis. Apart from ruling out existing proposals, a good model makes new predictions. We have undertaken precisely this type of detailed analysis in the mathematical follow-on paper, but we can already highlight one interesting prediction: we find that cargo richness increases as one moves from early to late stages of a maturation chain. In principle this is measurable by organelle proteomics, and therefore falsifiable.

Many successful models in physics and biology have been not predictive but explanatory, providing a unified and elegant resolution to a disparate set of known observations. It is often the case that complex and detailed model provide an illusion of rigor, but have outcomes that are rarely falsifiable. The best test of a model is to ask: are we getting out more than we put in? The simpler the model, the better the chance that the answer is “yes”.

## Author contributions

MT conceived the project. SM and MT designed the simulations. SM performed the simulations and analyzed the results. MT wrote the paper.
